# Implementation strategies for hospital-based probiotic administration in a stepped-wedge cluster randomized trial design for preventing hospital-acquired *Clostridioides difficile* infection

**DOI:** 10.1186/s12913-023-10350-9

**Published:** 2023-12-11

**Authors:** Lauren C. Bresee, Nicole Lamont, Wrechelle Ocampo, Jayna Holroyd-Leduc, Deana Sabuda, Jenine Leal, Bruce Dalton, Jaime Kaufman, Bayan Missaghi, Joseph Kim, Oscar E. Larios, Elizabeth Henderson, Maitreyi Raman, Jared R. Fletcher, Peter Faris, Scott Kraft, Ye Shen, Thomas Louie, John M. Conly

**Affiliations:** 1 Department of Community Health Sciences, Cumming School of Medicine, University of Calgary, Calgary, AB Canada; 2 O’Brien Institute of Public Health, University of Calgary, Calgary, AB Canada; 3 W21 Research and Innovation Centre, University of Calgary and Alberta Health Services, Calgary, AB Canada; 4 Department of Medicine, Cumming School of Medicine, University of Calgary and Alberta Health Services, Calgary, AB Canada; 5 Pharmacy Services, Alberta Health Services, Calgary, AB Canada; 6 Infection Prevention and Control, Alberta Health Services, Calgary, AB Canada; 7 Department of Microbiology, Immunology, and Infectious Diseases, Cumming School of Medicine, University of Calgary, Calgary, AB Canada; 8 Calvin, Phoebe, and Joan Snyder Institute for Chronic Diseases, University of Calgary and Alberta Health Services, Calgary, AB Canada; 9 Department of Pathology and Laboratory Medicine, Cumming School of Medicine, University of Calgary and Alberta Health Services, Calgary, AB Canada; 10 Department of Health and Physical Education, Mount Royal University, Calgary, AB Canada; 11 Department of Analytics, Alberta Health Services, Calgary, AB Canada; 12 AGW5 - Special Services Bldg, Foothills Medical Centre, 1403 29th Street NW, Calgary, AB Canada T2N 2T9

**Keywords:** Hospital-acquired *Clostridioides difficile* Infection, Probiotics, Protocol implementation, Focus group, Order entry

## Abstract

**Background:**

*Clostridioides difficile* infection (CDI) is associated with considerable morbidity and mortality in hospitalized patients, especially among older adults. Probiotics have been evaluated to prevent hospital-acquired (HA) CDI in patients who are receiving systemic antibiotics, but the implementation of timely probiotic administration remains a challenge. We evaluated methods for effective probiotic implementation across a large health region as part of a study to assess the real-world effectiveness of a probiotic to prevent HA-CDI (Prevent CDI-55 +).

**Methods:**

We used a stepped-wedge cluster-randomized controlled trial across four acute-care adult hospitals (*n* = 2,490 beds) to implement the use of the probiotic Bio-K + ® (*Lactobacillus acidophilus* CL1285®, *L. casei* LBC80R® and *L. rhamnosus* CLR2®; Laval, Quebec, Canada) in patients 55 years and older receiving systemic antimicrobials. The multifaceted probiotic implementation strategy included electronic clinical decision support, local site champions, and both health care provider and patient educational interventions. Focus groups were conducted during study implementation to identify ongoing barriers and facilitators to probiotic implementation, guiding needed adaptations of the implementation strategy. Focus groups were thematically analyzed using the Theoretical Domains Framework and the Consolidated Framework of Implementation Research.

**Results:**

A total of 340 education sessions with over 1,800 key partners and participants occurred before and during implementation in each of the four hospitals. Site champions were identified for each included hospital, and both electronic clinical decision support and printed educational resources were available to health care providers and patients. A total of 15 individuals participated in 2 focus group and 7 interviews. Key barriers identified from the focus groups resulted in adaptation of the electronic clinical decision support and the addition of nursing education related to probiotic administration. As a result of modifying implementation strategies for identified behaviour change barriers, probiotic adherence rates were from 66.7 to 75.8% at 72 h of starting antibiotic therapy across the four participating acute care hospitals.

**Conclusions:**

Use of a barrier-targeted multifaceted approach, including electronic clinical decision support, education, focus groups to guide the adaptation of the implementation plan, and local site champions, resulted in a high probiotic adherence rate in the Prevent CDI-55 + study.

**Supplementary Information:**

The online version contains supplementary material available at 10.1186/s12913-023-10350-9.

## Background

Hospital-acquired *Clostridioides difficile* infection (HA-CDI) is associated with considerable morbidity, mortality, and health care costs [1–3]. As such, prevention of HA-CDI is an important priority for patients who are hospitalized [4].

Use of systemic antibiotics is a common occurrence in hospital, and is a main risk factor for HA-CDI [5, 6]. A number of probiotics have been evaluated for the prevention of HA-CDI in randomized controlled trials (RCTs), however, the effect sizes vary possibly due to the type of probiotic used, the population studied (including baseline CDI rate), and study quality [7, 8]. In addition, it is unclear whether the results of available RCTs translate to real-world hospital settings given the restrictive nature of RCTs [9]. As such, the “Prevent CDI-55 +” study was initiated in Calgary, Canada in 2017, to evaluate the real-world use of a probiotic (Bio-K + ®, Laval, Quebec, Canada) to prevent HA-CDI in patients 55 years of age and older who were admitted to acute care hospitals and receiving systemic antimicrobials [10]. Each capsule of Bio-K + ® contained 50 billion colony-forming units of probiotic, and the organisms were *Lactobacillus acidophilus* CL1285®, *L. casei* LBC80R® and *L. rhamnosus* CLR2®.

The prescribing and timely administration of Bio-K + ® was important for the successful evaluation of the effectiveness of the probiotic for prevention of HA-CDI in the study population. As a result, the objective of this study was to identify potential barriers and facilitators to the successful implementation of the Prevent CDI-55 + study. We conducted focus groups and interviews throughout the Prevent CDI-55 + study to develop and adapt a multifactorial barrier- and facilitator-targeted strategy for the administration of Bio-K + ®.

## Methods

### Prevent CDI-55 + : study design and time frame

**Fig. 1:**
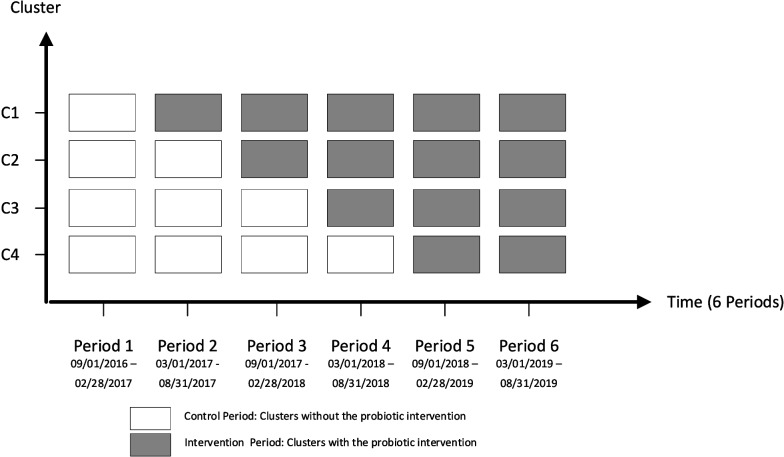
Prevent CDI-55 + study Basic Design. The clusters (C1, C2, C3, or C4) represent one hospital randomized to the four sequences where C = South Health Campus; C2 = Rockyview General Hospital; C3 = Peter Lougheed Centre; and C4 = Foothills Medical Centre. Each cluster included one or more 6-month control period(s) and an intervention minimum of one year. There was no transition time between periods

Briefly, Prevent CDI-55 + was a quasi-experimental, stepped-wedge cluster randomized design across four Alberta Health Services (AHS) acute-care adult hospitals (*n* = 2,490 beds in Calgary, Alberta, Canada) to implement the use of Bio-K + ® in patients ≥ 55 years of age who were receiving therapeutic antimicrobials with the objective to evaluate whether the real-world use of probiotics in these patients reduced the incidence of HA-CDI [10]. The study was initiated at the South Health Campus (SHC) starting March 1, 2017, the Rockyview General Hospital (RGH) starting September 1, 2017, the Peter Lougheed Centre (PLC) starting March 1, 2018, and the Foothills Medical Centre (FMC) starting September 1, 2018 [10]. Study follow-up ended on August 31, 2019 for all study sites. Based on the stepped-wedge design, hospitals served as their own controls until the study was initiated at their respective sites (Fig. 1). Patients in Prevent CDI-55 + were to be prescribed Bio-K + ® at the same time as their systemic antimicrobial prescription, to be continued until 5 days after the last dose of therapeutic antibiotic [10]. Adherence to probiotic prescribing was evaluated as the proportion of patients who were prescribed Bio-K + ® among all patients who received a newly prescribed therapeutic antibiotic while in hospital [10]. All units including surgical, medical, and intensive care units at each of the hospitals were included; hematology-oncology units, and patients with ileus or who were *nil per os* were excluded [10].

### Implementation interventions

To best support the uptake of probiotics for Prevent CDI-55 + , our implementation strategy was multifactorial and considered local site barriers to change. Site-specific discussion groups were conducted one month prior to the implementation start dates for each of the four sites. The aim of the discussion groups was to inform local implementation strategies, including targeting implementation interventions to potential barriers specific to each site. Each discussion group involved 6 to 12 individuals, and included Infection Prevention and Control professionals, infectious disease physicians, pharmacists, nurse educators, and key partners from surgery, medicine, and medical operations at each site. The resulting implementation interventions were made available to each site.

### Computerized clinical support tool

Acute care hospitals in Calgary use the electronic medical record system Sunrise Clinical Manager (SCM) for all patients admitted to hospital. SCM has the ability to build decision support tools to assist with the clinical management of patients. A clinical support tool was developed for Prevent CDI-55 + to ensure that when a prescriber entered an order for a systemic antimicrobial, they would receive an alert to also prescribe the probiotic with one click. The tool was built in October 2016, and piloted over four months prior to the initiation of Prevent CDI-55 + to identify and mitigate any issues. The attending physician and /or resident staff were generally responsible for the orders but they could also be placed by the pharmacist on behalf of the physician following consultation with the attending physician. As with any medication, the patient could refuse to take the prescribed probiotic. The targeted time frame of probiotic initiation was within 12 to 24 h following the initial administered dose of the antibiotic.

### Site champions

Infection prevention and control physicians at each study location and two study pharmacists were identified as site champions. They were active members of the study team, contributing to the study design and the development of the probiotic order in SCM. At the respective sites, they provided education to clinical staff on the study, supported local infection control professionals on the collection of the primary outcome as part of routine surveillance, and reported back any study site concerns back to the research team.

### Provider and organization education

Partner education was another important component for the uptake of this initiative. The targeted audience was prescribers of antibiotics, multidisciplinary health care teams including nurses and clinical pharmacists, and targeted organizational leaders, including site and executive leads. Prior to implementation at the first site, educational materials including education sessions, information packages, print and web-based materials, were developed by members of the research team. Educational sessions and materials were tailored to the targeted audience in order to help inform and reinforce a change in prescriber practice.

Education sessions were conducted at each participating site to inform local change in prescribing practice at the corresponding site. The sessions included presentations at key meetings (physician rounds, nurse education days, and site leadership meetings) and provided information on Prevent CDI-55 + , Bio-K + ® probiotics, and the effectiveness of probiotic use. Frequent presentations were conducted for the first three months at each site once the specific site started implementation, and continued as requested throughout the implementation period.

Posters, table cards (foldable cards placed at worksites convenient to the healthcare workers), and brochures containing information on Prevent CDI-55 + and the effectiveness of probiotic use were distributed at each site during the first month of implementation. In addition, web-based materials including an informational power-point presentation and a staff perspective video was available to prescribers and patients on the AHS website once all four sites had undergone implementation of the probiotic. The power-point presentation provided an outline of Prevent CDI-55 + , clinical findings on the effectiveness and safety of probiotics use, and the importance of reducing HA-CDI. The staff perspective video contained interview footage of consented health care providers and patients sharing their experience with CDI. The AHS website is freely available to AHS staff and the public (www.albertahealthservices.ca).

Educational material produced by the makers of Bio-K + ® was not used in this study or in the Prevent CDI-55 + study.

### Pharmacy services

To ensure the Prevent CDI-55 + study reflected real world practice, the probiotic was provided by inpatient pharmacy services at each site, and did not involve investigational pharmacy services. As a result, support for this project was provided by pharmacy services and nursing to ensure the probiotic was dispensed and administered to patients appropriately.

### Patient education

Patients included in Prevent CDI-55 + received a patient information package upon probiotic administration containing a brochure illustrating the initiative, the use of probiotics, and its effectiveness in preventing HA-CDI. Additionally, the package included information and findings from clinical studies on the effectiveness and safety of Bio-K + ® probiotics as an option for probiotic use, along with information about which local pharmacies carried Bio-K + ®.

All printed material provided in the patient information package was reviewed and approved by three CDI patients, a pharmacy sub-committee, AHS Communications, and the AHS Engagement and Patient Experience committee before the initial implementation start date.

### Implementation qualitative barrier and facilitator assessment

Focus groups were conducted to identify ongoing barriers and facilitators to the implementation of Bio-K + ® prescribing throughout the duration of the Prevent CDI-55 + study. A series of semi-structured focus groups were conducted with pharmacists, physicians, and nurses, to understand their experiences with implementing probiotics during the Prevent CDI-55 + study time frame. Volunteers were recruited using purposive sampling (selected wards or units where high rates of antibiotic prescribing occurred which were predominantly medical, surgical and critical care wards/units) via email, word of mouth by the site champions and research team, and through a recruitment poster displayed in each hospital, to ensure the participants were representative of the hospital units included in Prevent CDI-55 + . The first focus groups were conducted following initiation of the Prevent CDI-55 + study at the first study site, SHC (March 1, 2017), and subsequent focus groups were conducted at the additional study sites based on the initiation of the Prevent CDI-55 + study at each site (RGH: September 1, 2017; PLC: March 1, 2018; FMC: September 1, 2018). The timing of the focus groups at each site was planned to ensure adequate exposure to the Prevent CDI-55 + study at each respective site to allow for informed responses regarding the barriers and facilitators to initiation. Focus groups were conducted by two members from the research team, and the discussions were recorded, transcribed, and de-identified. The script used for the semi-structured interview is provided in Appendix 1.

### Qualitative analysis

The qualitative evaluation of the implementation of probiotics was conducted using two theoretical frameworks: the Theoretical Domains Framework (TDF) and the Consolidated Framework of Implementation Research (CFIR) [11, 12]. TDF and CFIR were used to ensure both individual behaviour and organizational behaviour were considered in the evaluation [13]. The links between the CFIR and the TDF are provided in Table 1. The coding frame was developed using a modified content analysis of the transcripts from the focus groups and an inductive approach to identifying themes that emerged. Coding was completed by a study team member, and the codes and relevant excerpts from the focus groups were discussed with other research team members to ensure agreement of the coding assignment. Themes and subthemes were similarly identified for both barriers and facilitators. Key CFIR and TDF domains and their alignment were identified based on frequency of mention during the focus groups, and were further described using themes and subthemes. Disagreements were resolved through discussion. NVivo 11 software was used to conduct the qualitative analysis.

**Table 1:** Individual and Organizational Behaviour Based on the Consolidated Framework for Implementation Research (CFIR) and the Theoretical Domains Framework (TDF) and their alignment [11, 12]

CFIR	TDF
Physical Capability	Physical Skills
Psychological Capability	Knowledge
	Cognitive & interpersonal skills
	Memory, attention & decision processes
	Behavioural regulation
Reflective Motivation	Professional/social role & identify
	Beliefs about capabilities
	Optimism
	Beliefs about consequences
	Intentions
	Goals
Automatic Motivation	Reinforcement
	Emotion
Physical Opportunity	Environmental context & resources
Social Opportunity	Social influences

### Ethics approval

Ethics approval to conduct this study was obtained from the Conjoint Health Research Ethics Board at the University of Calgary (REB16-1834). Specifically, a waiver of individual consent for in-hospital administration of probiotics to the patients in the quasi-experimental, stepped wedge, cluster- randomized controlled trial (Prevent CDI-55 +), was granted. However, patients received an information package upon Bio-K + administration containing information on the initiative, use of probiotics, their effectiveness in preventing CDI, and their safety. In addition, a waiver of consent was also granted for health information access, consistent with the provisions of the Health Information Act of Alberta. Consent was obtained at the time of agreement to participate by individuals in the focus groups. The information sheet that was provided to all focus group participants is provided in Appendix 2. The different facets of this study adhered to the most recent version of the Declaration of Helsinki.

## Results

### Implementation activities

A total of 340 educational sessions were provided at the four sites participating in the Prevent CDI-55 + study, and included more than 1,800 key partners and participants.

Adherence to the probiotic prescribing in hospital ranged from 76.9%—84.6% when stratified by study sites and time periods. When evaluating timing of probiotic administration, adherence within 48 h of antibiotic administration ranged from 60.2% to 71.4%, and within 72 h ranged from 66.7% to 75.8%.

### Focus groups

Two focus groups and 7 interviews (20 to 60 min in duration) were conducted with a total of 15 participants (1 to 6 participants per group). Of the 15 participants, 4 were registered nurses, 1 was a clinical nurse educator, 3 were physicians, and 7 were pharmacists. One focus group and 3 interviews took place at the SHC from May to August 2017, 3 interviews were conducted at the RGH between March and April 2018, 1 interview was conducted at the PLC in June 2019, and 1 focus group was conducted at the FMC in July 2019.

Themes and subthemes were identified across the CFIR and TDF behaviour change domains. Themes identified relating to the domain of environmental context and resources included process implementation and intervention characteristics. Identified barriers to process implementation were the alert system, medication time, and workload. Regarding the alert system, one participant stated: *“The alert fatigue is a well-known problem with SCM so I’m not sure there’s necessarily a way around it with this initiative—like I said unless you made them look a lot different than other alerts. I think, well a lot of times they do get ignored they are probably having some effect.”* In terms of medication time, a participant said: *“Well I think the gap is that people you schedule medications for 8am but you have several patients and so only one person truly gets them at 8am.”* Among the facilitators identified for process implementation, subthemes included patient teaching, order sets, and initiative communication. One participant commented: *“The more efficient ways to make it really efficient for the physicians, to not have several extra steps if they want to order it. So, having all that [Bio-K* + *®] in the order set with the antibiotic is really good for compliance so you don't have to think about it you just order it.”*

Subthemes of intervention characteristics that were identified as barriers included price, pill size, and post-discharge compliance. While Bio-K + ® was provided to patients in hospital, patients would have to purchase Bio-K + ® once they were discharged from hospital (to complete their five day post-antibiotic course), and staff were concerned that patients would be disinclined to purchase or unable to afford it, reducing post-discharge compliance. One participant noted: *“On discharge for some people especially like completing the protocol as it’s written. For a lot of people they’ll get it while they’re in hospital but will maybe step down their antibiotics to an oral antibiotic and they are ready to go home and only have 1–2 days of antibiotic left. So technically they have another 5–6 days of Bio-K* + *® to finish and they aren’t going to be able to get Bio-K* + *® at their nursing home.”* In addition, staff commented that the size of the Bio-K + ® capsule was a barrier: *“There’s lots of times they just outright refuse. I have had patients who’ve tried to take them and spit them back out because they can’t swallow them.”*

Knowledge barriers that were identified included health care provider education, literature, and evidence. Attending physicians were unsure if the medical residents had the same level of training and education on Prevent CDI-55 + , such as the inclusion criteria for patients. In addition, some participants were concerned about the evidence for probiotics: *“I know lots of physicians expressed that it’s controversial. Probiotics are a controversial field. Because it’s controversial it ranks low on their list of priorities as well sometimes.”* However, literature and evidence were also noted as a facilitator by a participant, in addition to patient education: *“I just say it's a probiotic that helps to counteract the effects of the antibiotics and to replenish the good bacteria in your body. And I say it's like eating yogurt but way stronger.”*

Barriers related to social and professional role and identity included the physician’s role, the nurse’s role, and the pharmacist’s role. Nurse and pharmacist participants noted that they could suggest to the physician to prescribe Bio-K + ®, however, they could not start a patient on Bio-K + ® independently. However, nurse and pharmacist participants also noted this as a facilitator to Bio-K + ® prescribing: *“I know at hand over I just I mindfully remember, I’ll pay attention to patients who are on antibiotics and right away I’ll ask if Bio-K* + *® needs to be started so during my shift I can make sure those are started. So that’s what encourages me to look into and get Bio-K* + *® started.”*

Table 2 lists the identified barriers, facilitators, and changes that were made to address the barriers and facilitators during the Prevent CDI-55 + study. Based on the results of the focus groups, a number of implementation initiatives were adapted to minimize potential barriers to the uptake of Bio-K + ® in Prevent CDI-55 + . The first adaptation was to the pop-up alert in SCM that reminded clinicians to prescribe Bio-K + ® when a therapeutic antibiotic was ordered. The alert was updated to make it more user friendly to ensure Bio-K + ® could be prescribed with one click. In addition, education for nursing staff was updated to address patients not being able to swallow Bio-K + ®; nursing staff was educated that the capsule could be opened and the contents added to food or liquid for administration. Lastly, to ensure patients could access Bio-K + ® after being discharged from hospital, patients were provided with a list of pharmacies that sold Bio-K + ®.

**Table 2:** Reported barriers, facilitators, and implemented changes

Theme	Reported Barrier or Facilitator	Implemented Change
Process implementation	Barrier: alert system, alert fatigue	Updated the originally developed alert that popped up when a therapeutic antibiotic was ordered to ensure Bio-K + ® could be ordered with one click
	Barrier: timing of probiotic administration	Timing of administration was modified wherever possible to minimize barrier
	Barrier: workload	Researchers attempted to minimize the impact to workload based on the changes listed in this table
	Facilitator: patient teaching	All patients received an information package on the Prevent CDI-55 + study
	Facilitator: Order sets	An alert was created within the electronic order entry system to ensure Bio-K + ® could be ordered with one click when a therapeutic antibiotic was ordered
	Facilitator: Initiative communication	Information regarding the Prevent CDI-55 + study, as listed in the methods section, was provided on a regular basis at each study site throughout the duration of the study
Intervention characteristics	Barrier: price of probiotic	No changes were made
	Barrier: probiotic capsule size	Nursing staff was provided education regarding opening the capsule and sprinkling the contents on food or in liquid for administration
	Barrier: post-discharge compliance	Patients were provided with a list of pharmacies that sold Bio-K + ®
Knowledge	Barrier: health care provider education	Ongoing health care provider education was provided during the Prevent CDI-55 + study
	Barrier: literature and evidence for probiotics	Information packages, and print and web-based materials were created and available to all AHS staff
Social and professional role	Barrier and facilitator: role of the health care providers	No changes were made

## Discussion

We created a multifaceted barrier-targeted intervention strategy to implement the Prevent CDI-55 + study across four adult hospitals in Calgary, Canada. Specifically, we conducted focus groups with front-line staff during the Prevent CDI-55 + study to identify ongoing barriers and facilitators for probiotic implementation. This information was then used to adapt the multifaceted intervention strategy accordingly.

We found the most commonly discussed barriers to Bio-K + ® implementation were related to the process of prescribing the probiotic, intervention characteristics including administration of Bio-K + ® and post-discharge compliance, along with knowledge relating to probiotic effectiveness for preventing HA-CDI and knowledge regarding Prevent CDI-55 + . Based on the identified barriers, we specifically modified the prescribing alert for Bio-K + ® in the electronic order entry system, updated nurse education with regards to the administration of Bio-K + ®, and ensured patients were aware of how to access Bio-K + ® after discharge from hospital. We also utilized multi-component targeted education strategies throughout the implementation phase at each site.

Previous studies that have evaluated the use of probiotics to prevent HA-CDI have reported low adherence to their respective study protocols. For example, a study by Trick and colleagues reported that only 26% of eligible patients received a probiotic in their study that was conducted in a 694-bed teaching hospital in Chicago, USA [14]. Similarly, Carstensen and colleagues reported a probiotic adherence rate of 44% in their study, and Cruz-Betancourt et al. reported a probiotic adherence rate of 39% [15, 16]. Lastly, adherence to probiotic prescribing has been discussed as an important component for quasi-experimental studies evaluating the impact of probiotics on HA-CDI [17]. The low adherence to probiotics seen in previous studies emphasizes the need for an adaptable multifaceted implementation strategy that considers barriers to implementation to ensure the effective uptake of probiotics, as was demonstrated in the in-hospital adherence to probiotics in the Prevent CDI-55 + study.

To our knowledge, this is the first study to evaluate barriers and facilitators related to the implementation of probiotics in a real-world effectiveness study. Conducting the focus groups throughout implementation allowed participants to have some prior exposure to the study and associated probiotic, better informing their responses related to implementation barriers and facilitators. In addition, feedback from the focus group participants during the Prevent CDI-55 + study allowed the study team to adapt the multifaceted implementation strategy to address identified ongoing barriers at each site. Lastly, the large number of partners engaged in the Prevent CDI-55 + study, and the many educational sessions conducted allowed us to reach staff on a regular basis to ensure identified barriers were addressed.

Our study is not without limitations. Specifically, there were barriers identified by participants that we were unable to address during the study, such as the concerns around adherence to Bio-K + ® after a patient was discharged from hospital. Although patient education was adapted to provide a list of pharmacies that carried Bio-K + ® at the time of discharge, the proportion of patients who completed the full course of Bio-K + ® post-discharge may have been low. Lastly, while only the general results of the adherence analysis are available in the Prevent CDI-55 + study, we did not examine the impact of each component of the multifactorial implementation strategy, or changes in the strategy over time, on adherence. This was due to the complexity of the stepped-wedge study design for the overall Prevent CDI-55 + study, limiting the ability to assess the change in the implementation strategy over time. However, we still note that the overall adherence to probiotic prescribing in the Prevent CDI-55 + study (76.9% to 84.6%) is numerically higher than the reported adherence to probiotics in previous hospital-based studies, ranging from 26 to 44% [14–16].

## Conclusions

Focus groups conducted throughout the Prevent CDI-55 + study helped identify ongoing barriers to implementation of probiotics targeted at reducing HA-CDI. These real-time data permitted the implementation interventions to be adapted to address these barriers. The result was higher adherence to probiotic prescribing, which has been a limitation to previous studies, allowing for less uncertainty in when assessing whether Bio-K + ® reduced rates of HA-CDI in the Prevent CDI-55 + study. Use of a 1-click order entry in SCM was considered a key component of the success of the implementation and should be considered for any implementation strategy for stewardship initiatives.

### Supplementary Information

**Additional file 1.**

**Additional file 2.**

## Data Availability

The datasets generated and/or analysed during the current study are not publicly available due to the data sharing policies of the University of Calgary and Alberta Health Services but are available from the corresponding author on reasonable request.

## Abbreviations

AHS: Alberta Health Services
CDI: *Clostridioides difficile* infection
CFIR: Consolidated Framework for Implementation Research
FMC: Foothills Medical Centre
HA-CDI: Hospital-acquired *Clostridioides difficile* infection
PLC: Peter Lougheed Centre
RCT: Randomized controlled trial
RGH: Rockyview General Hospital
SCM: Sunrise Clinical Manager
SHC: South Health Campus
TDF: Theoretical Domains Framework
