# Supplementary material for: Implementation strategies for hospital-based probiotic administration in a stepped-wedge cluster randomized trial design for preventing hospital-acquired Clostridioides difficile infection
Source: BMC Health Serv Res. 2023 Dec 11;23:1386. doi: 10.1186/s12913-023-10350-9 (PMC10714625; doi:10.1186/s12913-023-10350-9)
Supplement: Supplementary file 1 — Additional file 1. [file 12913_2023_10350_MOESM1_ESM.pdf]

Appendix 1: Semi-structured interview focus group script

Introduction:

Welcome and thank you for taking the time to join our discussion around your experience in the Prevent CDI-55+ initiative. My name is __________, I am a member of the W21C research team, and I will be the facilitator of this discussion. Our other team members include _________, ____________ and _________ . The project lead is Dr. John Conly, an infectious disease physician, and the project co-leads are Dr. Jayna Holroyd-Leduc, a geriatrician and Dr. Thomas Louie, a Professor in microbiology and an infectious disease physician and expert in C. difficile infection and treatment.

You have been invited here today because you are an important contributor to our initiative. Today we would like you to draw on your experiences around the barriers and challenges that you may have experienced as part of the Prevent CDI-55+ initiative. Thank you for your time and participation today.

There are no right or wrong answers. Keep in mind we are interested in both your positive and negative comments about the topics we discuss. We are interested in all comments, so we are taking detailed notes and also audio recording today's discussion, because we don't want to miss any of your comments and feedback. Please be assured that all of your comments will be kept confidential and any information you provide will be kept in a locked cabinet or stored in a password protected folder on a secure server, accessible only by members of the research team. We would also like to remind you that you are not obligated to share anything that you are not comfortable sharing. We would also ask that you respect the privacy of other participants by not discussing what is shared here outside of the session.

Feel free to get up and get refreshments if you would like.

Do you have any questions before we begin? The session will end at ______.

We are going to start with an overview of the topic.

The Prevent CDI-55+ quality improvement initiative aims to reduce C. difficile infections in admitted patients aged 55 years and older on systemic antibiotics. The use of probiotics to reduce C. difficile infections has promising implications for patient care and outcomes. Our study is testing the effectiveness of Bio-K+® probiotics in the "real world" acute care setting. This 3-year project has been started at South Health Campus, and will be implemented across all adult acute care hospitals in Calgary in a step-wise fashion.

We're currently exploring any barriers or challenges that you, the front-line staff, may have experienced during the implementation phase of the Prevent CDI-55+ initiative, or that you are currently experiencing. We hope to learn from your experiences and, where possible, incorporate your ideas and suggestions into the next phase of the project. We welcome you to share anonymized anecdotes about specific cases or patients as we talk today, as this will help us understand your perspectives better.

Ask the group:

1) Let's begin by going around the table to introduce ourselves. Please let everyone know your first name, and what role you have in health care.

2) By a show of hands, who is aware of the Prevent CDI-55+ initiative, and has been affected by it (e.g. through prescribing it for patients, educating staff, administering probiotic, or educating patients).

[Individuals with their hands raised] Because you have direct experience with our initiative, we are going to please ask that you complete a short survey.

Section 1

In this section, we would like to learn about what challenges you have experienced related to Prevent CDI55. We would like to discuss specific examples of challenges that you have experienced related to either prescribing, procuring, administering or educating about Bio-K+®.

3) What are the barriers you have experienced in trying to prescribe/provide eligible patients with probiotics?

- What could be done better?
- Do you have the necessary resources available to overcome these barriers?
- Do you have any suggestions to improve the process or available resources?

4) Are there procedures or ways of working that encourage or discourage you to follow the Prevent CDI-55+ protocol?

5) How does your work environment affect your ability to follow the Prevent CDI-55+ protocol?

Section 2

In this section, we would like to discuss how you communicate with your patients about the Prevent CDI55 initiative.

6) For patients on Bio-K+®, how do you educate them on the initiative?

- Do the patient education materials take into consideration the needs and preferences of the patients/families?

Summary and Wrap Up:

So now that we have talked about patient-centered care planning practices and the potential for digital solutions, I would just like to take a couple moments to summarize what we talked about today.

<Summarize Key Points>

Ask:

Is there anything else that anyone would like to add that we may not have captured in our discussions or in our summaries?

Thank you for coming and joining in our discussion today.

Thank them for coming, and invite to participate in future iterations.

General Probes:

- Does anyone see it differently?
- Are there any other viewpoints?
- Could you explain further?
- Could you give an example?

Debrief Facilitator and Assistants:

- What were the themes from the discussion?
- What were the overall impressions of the group?
- What was surprising?
- How was this group similar/different to other groups?
- Does anything need to be changed for next time?
