# Supplementary material for: Implementation strategies for hospital-based probiotic administration in a stepped-wedge cluster randomized trial design for preventing hospital-acquired Clostridioides difficile infection
Source: BMC Health Serv Res. 2023 Dec 11;23:1386. doi: 10.1186/s12913-023-10350-9 (PMC10714625; doi:10.1186/s12913-023-10350-9)
Supplement: Supplementary file 2 — Additional file 2. [file 12913_2023_10350_MOESM2_ESM.pdf]

UNIVERSITY OF CALGARY

Dr. John Conly, MD
Dr. Jayna Holroyd-Leduc, MD
Dr. Thomas Louie, MD

W21C Research and Innovation Centre
Cumming School of Medicine
3280 Hospital Drive, NW
GD01, TRW Building
Calgary, AB
Phone: 403.210.7450
Email: cdi55@ahs.ca

CONSENT FORM

TITLE: Probiotics to Reduce *Clostridium difficile* Infection and Antibiotic Associated Diarrhea in Hospitalized Older Adult Patients Receiving Antibiotics

INVESTIGATORS: Dr. John Conly, Dr. Jayna Holroyd-Leduc, and Dr. Thomas Louie

SPONSOR: Alberta Innovates

This information sheet is only part of the process of informed consent. It should give you the basic idea of what the research is about and what your participation will involve. If you would like more detail about something mentioned here, or information not included here, please ask. Take the time to read this carefully and to understand any accompanying information.

BACKGROUND

Prevent CDI55^{+} is a 3-year quality improvement initiative that aims to reduce *Clostridium difficile* infection in hospitalized patients through the use of probiotics, specifically Bio-K+. This initiative will be implemented across all adult care hospitals within Calgary. The success of this project is dependent on stakeholder engagement, including prescribers of antibiotics, multi-disciplinary healthcare teams looking after these patients, including clinical pharmacists; the organization (more specifically, hospitalists, nurse educators, site and executive leads); and the patients themselves. We are therefore seeking input from the key stakeholders to identify any challenges or barriers to implementation that may prevent a successful uptake of the initiative. Focus groups and interviews are useful tools often employed by researchers to learn a range of perspectives and opinions surrounding a particular topic. We are therefore performing targeted semi-structured interviews/focus groups to identify areas that may be improved during the implementation phase of this initiative.

WHAT IS THE PURPOSE OF THE STUDY?

The purpose of the focus group is to identify any barriers or challenges related to the implementation of the Prevent $CDI55^+$ initiative. As an end-user of this project, we are interested to directly learn from you, and hear about your experiences during any phase of the project. Our aim is to incorporate your feedback to future modifications that will enhance the overall implementation protocol, leading to improved adherence and user-experience.

WHAT WOULD I HAVE TO DO?

You will be required to participate in one (1) focus group, lasting no more than 30-minutes. Your level of participation is optional, however we do encourage you to contribute to the discussion so that we can learn as much as possible from your experience and perspective. An experienced researcher from W21C will commission the session, and you will asked to generally comment in three areas: 1) your experience with *Clostridium difficile* as a professional, 2) challenges experienced during the implementation of the Prevent CDI55+ initiative (*i.e*., procedural, environmental, education, etc.), and 3) Prevent CDI55+ patient education. The questions will be asked in a semi-structured nature, allowing for flexibility by the researcher administering the questions. You may also be asked to complete a short survey, requiring 3-5 minutes of your time.

WHAT ARE THE RISKS?

There is little risk to you for participating in this focus group. As the forum will be a group session, your identity within the group cannot be kept confidential. We do ask everyone participating in the session to respect the privacy of others, and not discuss what is shared outside of the session, however we cannot guarantee that other members of the group will keep contributions confidential. We will record (audio only) conversation during the session so that researchers can later retrieve, review and clarify comments and feedback if necessary.

WILL I BENEFIT IF I TAKE PART?

If you agree to participate in this study, there may or may not be a direct benefit to you. The information that we will obtain from the focus group will be used to direct changes for improvement within the Prevent $CDI55^+$ initiative.

DO I HAVE TO PARTICIPATE?

Participation in this focus group is voluntary, and you may withdraw your consent at any time with no repercussions to you. If you withdraw from the study, the data collected to the point of

withdrawal will still be used, as we cannot remove your individual contributions from the group discussion. If you decide to participate you have the right to ask any questions regarding the study at any time.

WILL I BE PAID FOR PARTICIPATING, OR DO I HAVE TO PAY FOR ANYTHING?

Participating in this focus group will be at no cost to you. If you must pay for parking to attend the focus group, this cost will be reimbursed.

WILL MY RECORDS BE KEPT PRIVATE?

If you participate in this study, the information that we obtain from you will be kept strictly confidential in locked cabinets and in authorized electronic data form, and will be used for research purposes only. The transcripts and audio recordings may be reviewed by W21C research personnel. No record bearing your name will be provided to anyone else except authorized representatives from the University of Calgary and the Conjoint Health Research Ethics Board for quality assurance purposes. You will not be identified as an individual in any report coming from this study.

AGREEMENT TO PARTICIPATE

Your decision to attend and participate in this focus group will be interpreted as an indication of your agreement to participate. In no way does this waive your legal rights nor release the investigators, or involved institutions from their legal and professional responsibilities. You are free to withdraw from the study at any time.

If you have further questions concerning matters related to this research, please contact:

Dr. John Conly (403) 944-8090

or

Dr. Jayna Holroyd-Leduc (403) 944-1771

If you have any questions concerning your rights as a possible participant in this research, please contact the Chair of the Conjoint Health Research Ethics Board, Research Services, University of Calgary, 403-220-7990.

The University of Calgary Conjoint Health Research Ethics Board has approved this research study.
